# Insect herbivory differentially affects the behaviour of two pollinators of *Brassica rapa*

**DOI:** 10.1007/s00442-025-05777-2

**Published:** 2025-08-14

**Authors:** Hanneke A. C. Suijkerbuijk, Erik H. Poelman

**Affiliations:** Laboratory of Entomology, Wageningen University & Research, Wageningen, The Netherlands

**Keywords:** Bumblebee, Butterfly, Herbivore-induced response, Plant reproduction, Plasticity, Pollination

## Abstract

Pollinator behaviour during floral visits shapes plant mating opportunities, influencing the likelihood of outcrossing versus selfing. These behaviours may be affected by plant responses to antagonists such as insect herbivores. This study aimed to better understand how insect herbivory affects visitation behaviour of two pollinator community members of field mustard (*Brassica rapa*): the buff-tailed bumblebee (*Bombus terrestris*) and the large cabbage white butterfly (*Pieris brassicae*)*.* In field tent experiments, we allowed caterpillars of *P. brassicae* to develop from leaf-feeding early instars to flower-feeding late instars and assessed pollinator response to undamaged and damaged plants during 10-min observations. We recorded behaviours with potential effect on outcrossing: (first) choice and overall plant preference, plant visit duration, number and duration of flower visits per plant, and distance travelled between plant visits. Bumblebees visited more plants and flowers, but spent less time per flower than butterflies did. Bumblebees often travelled to neighboring plants, whereas butterflies often flew longer distances to reach a next plant. Herbivory by large florivorous caterpillars altered butterfly plant choice-mostly towards plants with caterpillars, but not their behaviour on the plant. Bumblebees did not alter their choices, but changed their behaviour on the plant: they visited more flowers on plants with herbivory. However, herbivory by small leaf-feeding caterpillars did not affect the behaviour of either pollinator. Our results show that pollinator community members respond differently to herbivory and that this response changes over time, potentially affecting plant mating patterns.

**Supplementary Information:**

The online version contains supplementary material available at 10.1007/s00442-025-05777-2.

## Introduction

Plants live in a dynamic community alongside other plants, antagonists such as insect herbivores, and mutualists like pollinators and natural enemies of herbivores. The composition of these communities is highly variable throughout the season and its assembly is determined by direct and indirect interactions among community members (Mertens et al. 2024; Petanidou et al. 2008). For example, defence responses to deal with insect herbivore attack affect how plants maintain interactions with beneficial pollinators (Kessler et al. 2011).

Pollinator behaviour is a key component in pollen distribution and, therefore, plant mating opportunities (Mitchell et al. 2009; Suijkerbuijk et al. 2025). Behaviours that determine where picked-up pollen is deposited include choices of which plant to visit, how many flowers to visit of an individual plant before moving to a different plant, and the distance travelled between plant visits. For example, subsequent visits to flowers on the same plant can increase selfing in self-compatible plants (Karron et al. 2009), or loss of pollen (pollen discounting) and clogging of stigmas in self-incompatible plants (Barrett 2003; Minnaar et al. 2019). The duration of flower visits might increase the amount of pollen picked up or deposited by the pollinator (Kudo 2003). The pattern in which pollinators travel also matters: their visitation sequence determines which plants will have the opportunity to mate with one another (Lysenkov 2009). Ultimately, pollinators create opportunities for mating through facilitated self- or cross-pollination.

Different members of the pollinator community contribute differently to plant reproduction, due to their abundance, life history or behaviour (Adler and Irwin 2006; Ne’eman et al. 2010; Willcox et al. 2017). Some pollinators, such as bumblebees and honeybees, collect pollen for their offspring, which leads them to visit many plants in a very efficient manner. However, grooming and pollen collection by these pollinators also results in a loss of potential outcrossing to plants, as pollen collected for the larvae will not reach any conspecific plants (Minnaar et al. 2019). Other pollinator species, such as syrphid flies or butterflies, mainly visit flowers to sustain themselves with food and are not location-bound to a hive (Doyle et al. 2020). As a result, they may visit fewer plants or fewer flowers per plant and carry pollen over larger distances.

Pollinator behaviour is also affected by plant interactions with other species, such as insect herbivores (Haas and Lortie 2020). Negative effects occur when flower-feeding by herbivores decreases pollinator attraction by flower damage, or by reducing the number of flowers presented (McCall and Irwin 2006; Strauss et al. 1996). Additional effects of herbivory on pollinator behaviour occur through plant-mediated effects. Herbivore-induced changes that affect pollinator attraction and behaviour include changes in floral colour or UV-radiance, plant volatile emission or the quantity or quality of plant rewards (Rusman et al. 2019a, b; Schiestl et al. 2014; Stevenson et al. 2017). The changes in plant phenotype also depend on herbivore feeding location. For example, leaf-feeding can have different outcomes than flower-feeding (Rusman et al. 2019a, b).

Lastly, not all pollinator community members will respond in the same way to herbivory (Rusman et al. 2019a, b), since they have different needs and preferences. For example, bumblebees that collect pollen are likely to respond to decreased pollen quality (Ruedenauer et al. 2016), whereas pollen quality may not be relevant to nectar-collecting butterflies. When bumblebees encounter low reward quality, they may leave the plants sooner, reducing facilitated selfing and increasing the likelihood of outcrossing if their next visit is to a conspecific plant. How feeding damage by insect herbivory on leaves or flowers affects behaviour of different pollinators and their flower visitation patterns in a plant community is understudied.

In this study, we unravel how herbivory influences pollinator behaviour, and whether effects depend on pollinator identity. We also explore how the location of herbivory (damage to leaves vs. floral tissue) shapes outcomes. We investigate behaviours that potentially affect plant mating patterns: plant preference, plant and flower visitation frequency and duration, and the distance travelled between plants. We compare two different pollinator community members of field mustard (*Brassica rapa*)*:* buff-tailed bumblebees (*Bombus terrestris*) and large cabbage white butterflies (*Pieris brassicae*). We hypothesize that leaf and floral herbivory affect pollinator behaviours differently. As florivory causes direct damage to floral tissue, we expect the response to florivory to be stronger than the response to folivory. Additionally, the presence of herbivores in the flowers adds a visual cue to pollinators. We expect different responses in bumblebees and butterflies, as they have different foraging requirements with bumblebees foraging for nectar and pollen to feed their colony and butterflies to feed on nectar to sustain their own energy needs.

## Methods

### Plants and insects

Experiments were conducted using a wild accession of *B. rapa*, grown from seeds collected from populations near Wageningen in 2016. Seeds were cold treated at 4 °C for one week to synchronize and accelerate flowering. After this vernalization, plants were grown in a greenhouse for 5 weeks (21 °C ± 2 °C, 50–70% relative humidity, L16:D8). Seedlings were grown first in soil cubes and subsequently transferred to larger pots (ø23 cm, 5L) filled with ‘Lentse potgrond’ potting soil. Hereafter, plants were transferred to the experimental outdoor tents and grown for two more weeks.

The large cabbage white (*Pieris brassicae*) caterpillars and adults were obtained from the central rearing facility at the Laboratory of Entomology, where they are reared on Brussels sprouts (*Brassica oleracea* var. *gemmifera*) at 21 °C ± 2, 50–70% RH, and a L16:D8 photoperiod. Caterpillars in their first larval instar (L1) were used for induction treatments. The life history of *P. brassicae* makes it ideal for the study of pollinator responses to folivory and florivory. *Pieris brassicae* is a gregarious caterpillar and specialist herbivore of Brassicaceae plants. The first instar caterpillars feed exclusively on the plants leaves, corresponding with the first week of each experimental round in our study. Depending on temperature, after about 5–7 days, the larvae start to feed as florivores (Lucas-Barbosa et al. 2014; Smallegange et al. 2007). Therefore, we consider the treatment in the first week of each experiment as folivory, and the second week as florivory. The caterpillars in florivorous stage make substantial damage to the flowers and are also very well visible on the flower branches by their aposematic colouration. Only male *P. brassicae* adults were used as pollinators to prevent egg-laying on experimental plants. Adults were kept without food and used on the second day after emergence from the pupae. Buff-tailed bumblebee (*Bombus terrestris*) are common polylectic pollinators that include several Brassicaceae plants in the range of flowers they visit for nectar and pollen collection. They live in a colony of up to hundreds of individuals. The hives containing small colonies were obtained commercially (Natupol Smart, Koppert Biological Systems, The Netherlands).

Experimental set-up

The experiment was replicated three times, in experimental rounds of two weeks each. For each of the three experimental rounds, the 24 potted plants were placed in four rows of six plants, spaced one meter apart, in a large outdoor mesh tent of 12m × 12 m. As floral display size influences pollinator attraction, plants with a similar number of open flowers were selected for the experiment. Alternating plants were induced with *P. brassicae* caterpillars for the herbivory treatment, in a chessboard pattern. Plants were placed on dishes to facilitate watering and hamper caterpillar movement between plants. Eight L1 caterpillars were placed on the lower leaves of each *B. rapa* plant in the induction treatment. Each experimental round lasted two weeks, during which the caterpillars grew up to the 5th larval instar. Around the start of the second week, depending on temperature, the caterpillars started to move higher up the plant to feed on the flowers. Twice per week, control plants were checked for caterpillar presence. Caterpillars were found not to move between treatments. At the same time, caterpillar numbers and location on the induced plants were recorded. Not all individuals survived the transfer to the experimental plants, likely due to the tougher outdoor conditions compared to the rearing conditions, and the small size of the L1 caterpillars In case of reduced caterpillar numbers on treated plants, more caterpillars of similar larval stage were added to keep eight larvae on the plant.

Behavioural observations

Behavioural observations were carried out between 9:00 and 18:00 on weekdays, provided the temperature was above 18 °C and there was no rain. All measurement were carried out between 19 June and 28 July 2023. Individual pollinators were visually tracked one-by-one for 10 min while they were free to visit as many plants in the tent as they liked. Behaviours were live-tracked by the researcher, and scored on a tablet equipped with PocketObserver software (Version 15.0.1200, Noldus Information Technology B.V., The Netherlands). With this software, the researcher scores the current behaviour, while the software keeps track of the duration. Scored behaviours were: (1) visits to individual experimental plants (numbered 1–24); (2) visits to individual flowers, nested within plant visits; (3) flights between plant visits Butterflies (*N* = 170) were released one-by-one. Each individual butterfly was released by hand from the same position. The behavioural recording started directly after release. For bumblebees (*N* = 194) the hive was opened allowing 4–5 bees to exit. The bumblebee hive was located 2 m in front of the middle of the first row of six plants. Bumblebee recordings were started as soon as the first bee started interacting with the experimental plants. During this recording, we missed the first choices of the other bees. Therefore first choice was not included in the bumblebee metrics and we decided not to deepen analyses of how first flower visits altered the visitation behaviour of the pollinators. The different bees, recognizable by for example body size, were recorded one-by-one until they re-entered the hive after about 30–40 min of foraging. Recordings of each individual lasted 10 min, or shorter in case (1) the pollinator was unresponsive for 60 consecutive seconds or (2) the observer lost track of the individual or (3) when a bumblebee finished a foraging bout and re-entered the hive. Bumblebee and butterfly measurements were carried out intermittently, switching between pollinator species after 4–5 observations per species.

### Data analysis

Initial data processing was done using Observer XT software (version 15.0.1200). We extracted the number of (induced and control) plants visited per pollinator observation and the total observation duration. To compare pollinator behaviours on induced versus control plants the software calculated the mean, minimum, maximum and total number and duration of plant and flower visits for each plant within the observation. All analyses were performed using R (Version 4.4.1, The R Foundation for Statistical Computing). Since the experiment was repeated three times, the experimental round (1–3) was included in all models. Each experimental round spanned two weeks: in the first week the caterpillars were still on the leaves, and in the second week they started flower feeding. Therefore, ‘caterpillar feeding stage’ was included in the models to represent the response to leaf-feeding (folivory) versus flower-feeding (florivory). All model tests were performed using the glmmTMB and car packages (Brooks et al. 2017; Fox and Weisberg 2019) and assessed for fit using QQ and residuals plots in the DHARMa package to visually assess the assumptions of normality and homoscedasticity of the residuals (Hartig 2022). In some cases outliers in the data made the model fit more challenging, and in these cases data was log-transformed to improve model fit as described below. Post-hoc testing was performed using the emmeans package (Lenth 2024). *T *tests were performed using base R functions. Plots were generated using the ggplot2, ggpattern and cowplot packages (FC et al. 2024; Wickham 2016; Wilke 2024).

#### Analysis of choice behaviour

First choice was analysed for *P. brassicae* butterflies only, as these observations started from the release point. No data were available for butterflies in the first week of the initial experimental round, due to unavailability of the species for that experimental round. Therefore, the overall model was constructed to include only the second and third experimental round. First choice was either for a ‘control plant’ or ‘herbivory’ plant. This Generalized Linear Model (GLM) had ‘first choice’ as the response variable ‘caterpillar feeding stage’ (folivory or florivory) and ‘experimental round’ (1–3) as fixed explanatory variables, using a binomial distribution. The choices for plants with or without caterpillars made during the entire observation were analysed for both butterflies and bumblebees. As butterflies and bumblebees differed in visitation rate, and not all individuals could be tracked for a full 10 min, for each individual we calculated the fraction of choices for plants with caterpillars, rather than the absolute number of visits to plants with herbivores. We included only the observations where the individual visited at least two plants, or that lasted the full 10 min. The model included ‘fraction of choices for herbivory’ as response variable, and ‘pollinator species’ (bumblebee or butterfly), ‘experimental round’ and ‘caterpillar feeding stage’ as fixed explanatory variables, with a normal distribution.

#### Analysis of visitation behaviour

Visitation behaviour included three metrics: time spent per plant, number of flowers visited per plant, and the time spent per flower. Behaviours were averaged per individual pollinator and per treatment. Similar linear mixed models (LMMs) were constructed for each behaviour. The response variables for the three models were (1) the log of the mean time spent per plant visit; (2) the log of the number of flower visits per plant, and (3) the log of the time spent per flower visit. The explanatory variables included in the model, with full interaction terms, were ‘treatment’ (indicating a visit to a plant with or without caterpillars), ‘pollinator species’ (bumblebee or butterfly), ‘experimental round’ (1–3) and ‘caterpillar feeding stage’ (folivory or florivory). While experimental round was not central to the study question, it was consistently found to have a large effect and was therefore included as a fixed effect rather than a random factor. Additionally, model fit was found to be better when round was included as a fixed rather than random factor. Individual pollinator identity (observation number) was regarded as a random factor. The log-transformed data were normally distributed. For time spent per flower, no appropriate fit could be found to analyse the data for both pollinators together, as the residual variation differed too much between the species. Data were, therefore, analysed separately for each pollinator.

#### Analysis of spatial behaviour

To analyse spatial behaviour, a matrix was constructed to calculate distances between all plants, and subsequently the average and total distances travelled per individual. To assess treatment effect, the average distance travelled to the next plant after encountering either a control plant or plant with caterpillars was calculated and used for further analysis. A GLMM with lognormal distribution was constructed with distance travelled as the response variable. Included as fixed explanatory variables with full interaction terms were ‘treatment’, ‘pollinator species’,’caterpillar feeding stage’ and ‘experimental round’. Included as a random factor was, ‘individual pollinator identity’.

## Results

### Choice behaviour

#### First choice

**Fig. 1:**
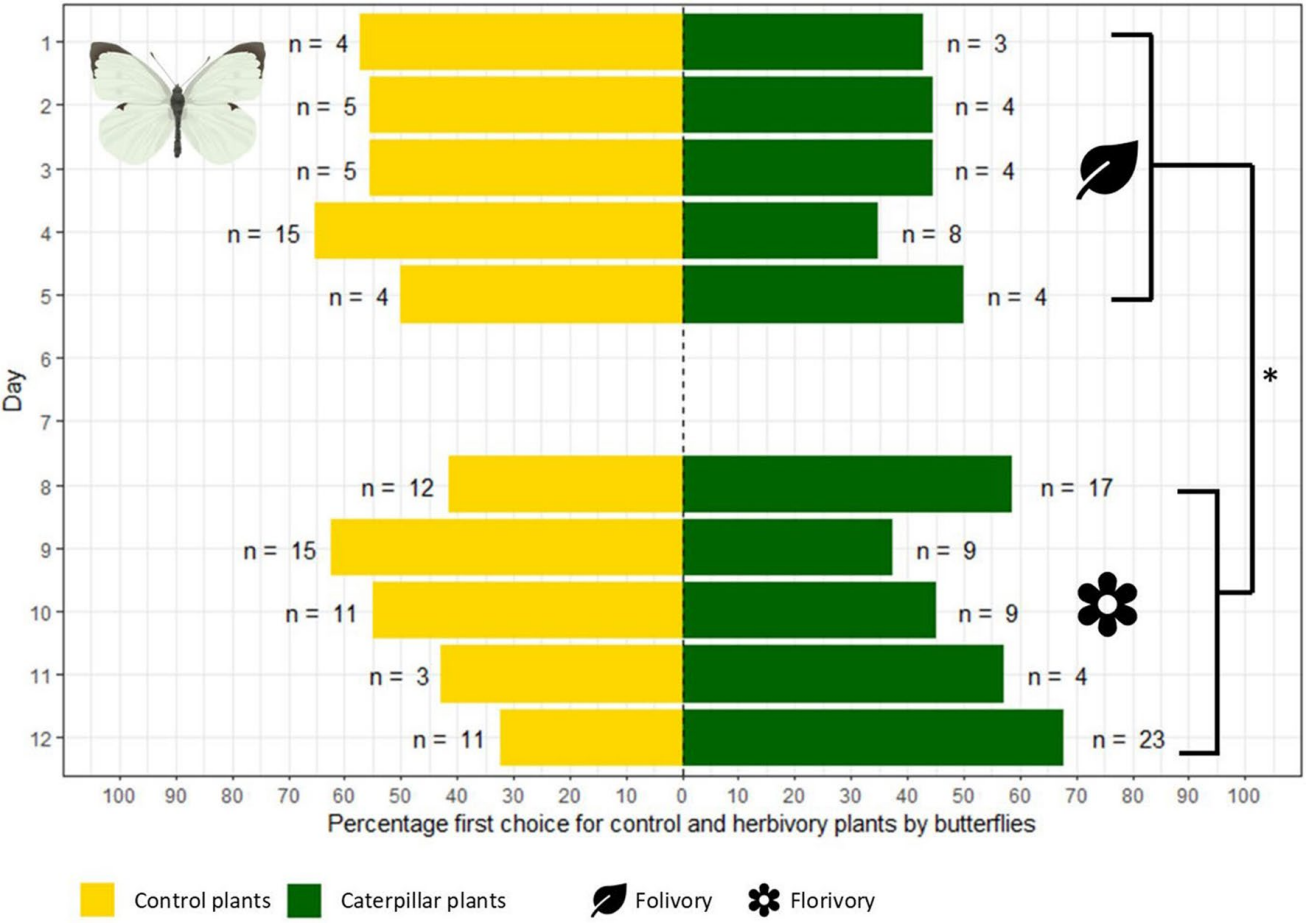
First choice of *Pieris brassicae* butterflies. Preference of each individual butterfly plotted for each day across the three two-week experimental rounds. Day 1 marks the induction of plants with *Pieris brassicae* caterpillars. During the first week (Days 1–5) caterpillars were mostly feeding on the leaves, and shifted to feeding on the flowers in week 2 (Days 8–12). Yellow bars (left) indicate the percentage of individuals with a first choice for control plants. Green bars (right) indicate the percentage of individuals with a first choice for plants with herbivores. N represents the number of individuals. Significance code: *p < 0.05

Taken together over all three rounds of measurement, *P. brassicae* butterflies showed no visitation preference for plants with or without caterpillars (50% or 85 out of 170 butterflies choose control plants). However, butterfly choices were affected by caterpillar age: the first choice differed between the first week—when the caterpillars fed as folivores—and second week, when the caterpillars started to feed on the flowers (GLM, *χ*^2^ = 5.134, *df* = 1, *p* = 0.0234, Fig. 1). Post-hoc analysis revealed that this was mostly due to a strong effect in the second experimental round (Tukey, *p* = 0.008, Figure S1b), whereas the effect was not clear in the third experimental round (Tukey, *p* = 0.460, Figure S1c). In the second experimental round, 73% of butterflies choose plants with florivorous caterpillars first (29 out of 40). On the final day of this experimental round, all butterflies (*n* = 10) even choose to land on a plant with herbivory first. No significant preference for control plants or those with florivorous caterpillars was found in experimental rounds 1 and 3 (Table S1). In the first experimental week, measured in experimental rounds two and three, butterflies showed no clear preference for control or plants with folivorous caterpillars. First choice was not assessed for bumblebees (see Methods).

#### Choices during the observations

Bumblebees and butterflies differed in their visitation rate (Welch’s *t *test, *t* = 3.40, *df* = 142.38, *p* < 0.001, Supplementary Table S2, Figure S2a). On average, bumblebees visited 9.69 (± 5.87) plants whereas butterflies visited 6.93 (± 4.93) plants during the 10-min observation (Supplementary Table S2).

**Fig. 2:**
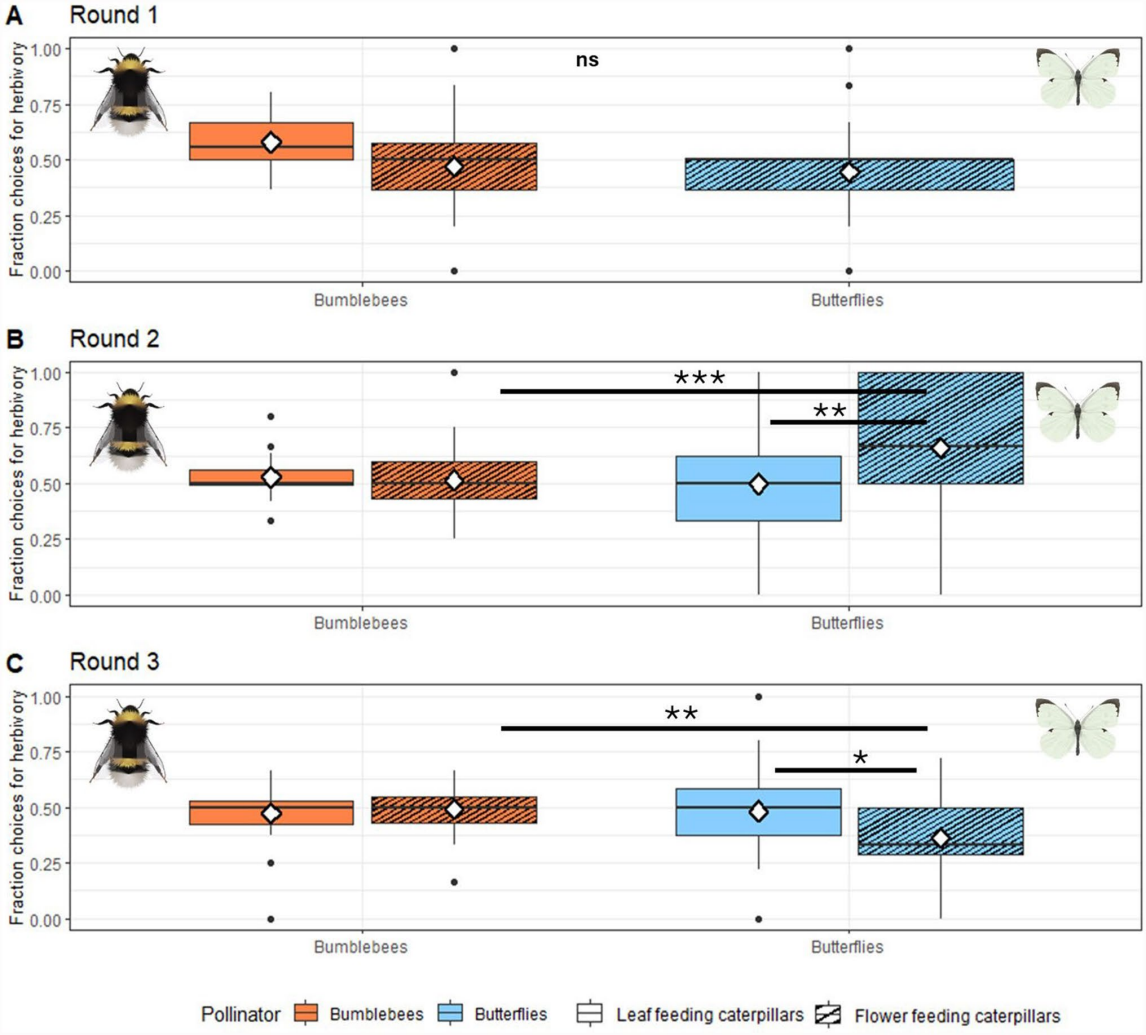
Fraction of choices for plants with herbivory per experimental round (**A–C**) and pollinator. **A–C** Fractions of choice of caterpillar-induced plants over control plants for bumblebees (*Bombus terrestris;* orange) and butterflies (*Pieris brassicae;* blue). Blank boxes indicate folivory; striped boxes indicate florivory. Diamonds indicate the mean values. Significance codes: *p < 0.05; **p < 0.01

Throughout the whole experiment, both bumblebees and butterflies visited a roughly equal fraction of control and herbivory plants. On average, the fraction of plant visits to herbivory plants was 0.5 (± 0.15) for bumblebees, and 0.49 (± 0.25) for butterflies (Supplementary Table S4, Supplementary Figure S3). The response to herbivory depended on the pollinator, caterpillar feeding location and experimental round with significant interactions among these factors (GLM, three-way interaction, *χ*^2^ = 10.169, *df* = 1, *p* = 0.001). In the first experimental round there was no difference in behaviour between bumblebees and butterflies (Tukey, *p* = 0.589, Fig. 2a)*.* In the second and third experimental round the pollinators clearly differed in their preference for plants with florivorous caterpillars (Tukey, *p* < 0.001 and *p* = 0.002 for rounds two and three respectively, Fig. 2b, 2c). In the second experimental round, butterflies visited a larger fraction of plants with florivorous herbivores, compared to plants with folivorous herbivores (Tukey, *p* = 0.001). Interestingly, in the third experimental round they showed the opposite preference: the butterflies visited plants with florivorous caterpillars less often than they visited plants with folivorous caterpillars (Tukey, *p* = 0.011). Bumblebees did not change their preference to plants with leaf- or flower feeding caterpillars in experimental rounds two and three.

### Visitation behaviour

#### Time spent per plant

**Fig. 3:**
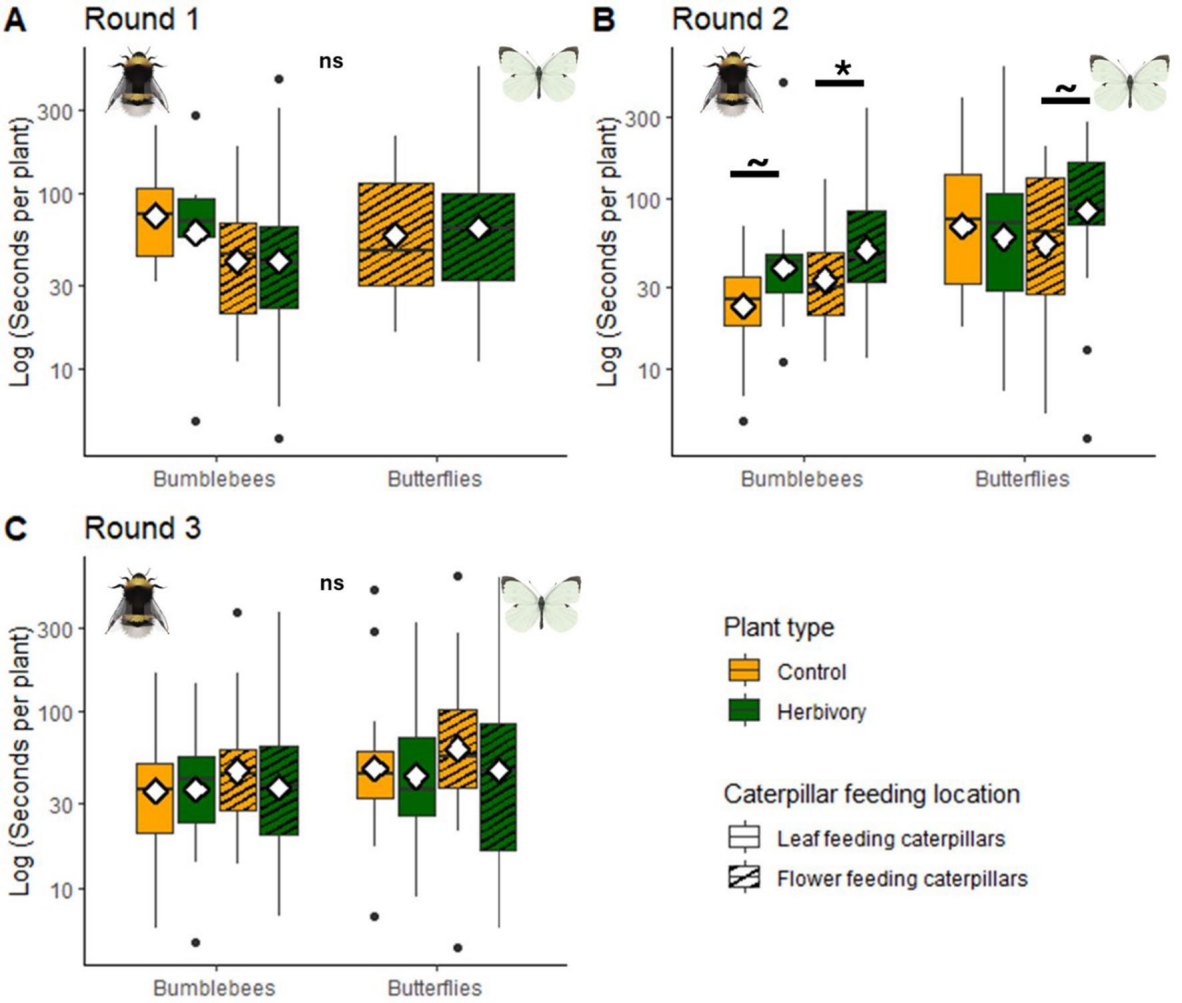
Time spent per plant with or without herbivory, by bumblebees and butterflies*. A–C* Experimental rounds 1–3. Yellow boxes indicate control treatments for folivory (blank) and florivory (striped). Green boxes indicate herbivory treatments of folivory (blank) and florivory (striped). Significance codes: ~ *p* < 0.1; *p < 0.05

On average, butterflies spent 92 s (± 106 s) per plant visit, which was significantly longer than bumblebees, that spent on average 56 (± 61) seconds on a plant visit (LMM, Pollinator main effect, *χ*^2^ = 29.64, *df* = 1, *p* < 0.001, Figure S2b). *Bombus terrestris* bumblebees on average spent 10.62 s more on plants with caterpillars, compared to control plants (mean = 61.39 ± 73.23 and mean = 50.77 ± 45.50, respectively; Supplementary Table S5). *Pieris brassicae* butterflies also spent on average slightly more time on herbivory plants—i.e. 13.39 s—compared to control plants (mean = 98.90 ± 120.02 and 84.92 ± 90.00, respectively). However, the variation was large and the overall effects were not significant. For time spent per plant, the response to herbivory depended on experimental round (Treatment * Experimental round interaction, LMM, *χ*^2^ = 9.87, *df* = 2, *p* = 0.007, Fig. 3). In the first experimental round, there was no significant difference in the time spent on control versus herbivory plants in either week for either pollinator. In the second experimental round, bumblebees spent more time on plants with caterpillars: the effect was not significant when the caterpillars were feeding on leaves (Tukey, *p* = 0.072), but significant when the caterpillars were feeding on flowers (Tukey, *p* = 0.03). During the second experimental round, butterflies also tended to spend more time on plants with flower-feeding caterpillars compared to control plants, but this effect was not significant (Tukey, *p* = 0.063). In the third experimental round, there was no effect of herbivory or caterpillar feeding location on visitation duration of either pollinator.

#### Number of flower visits per plant

**Fig. 4:**
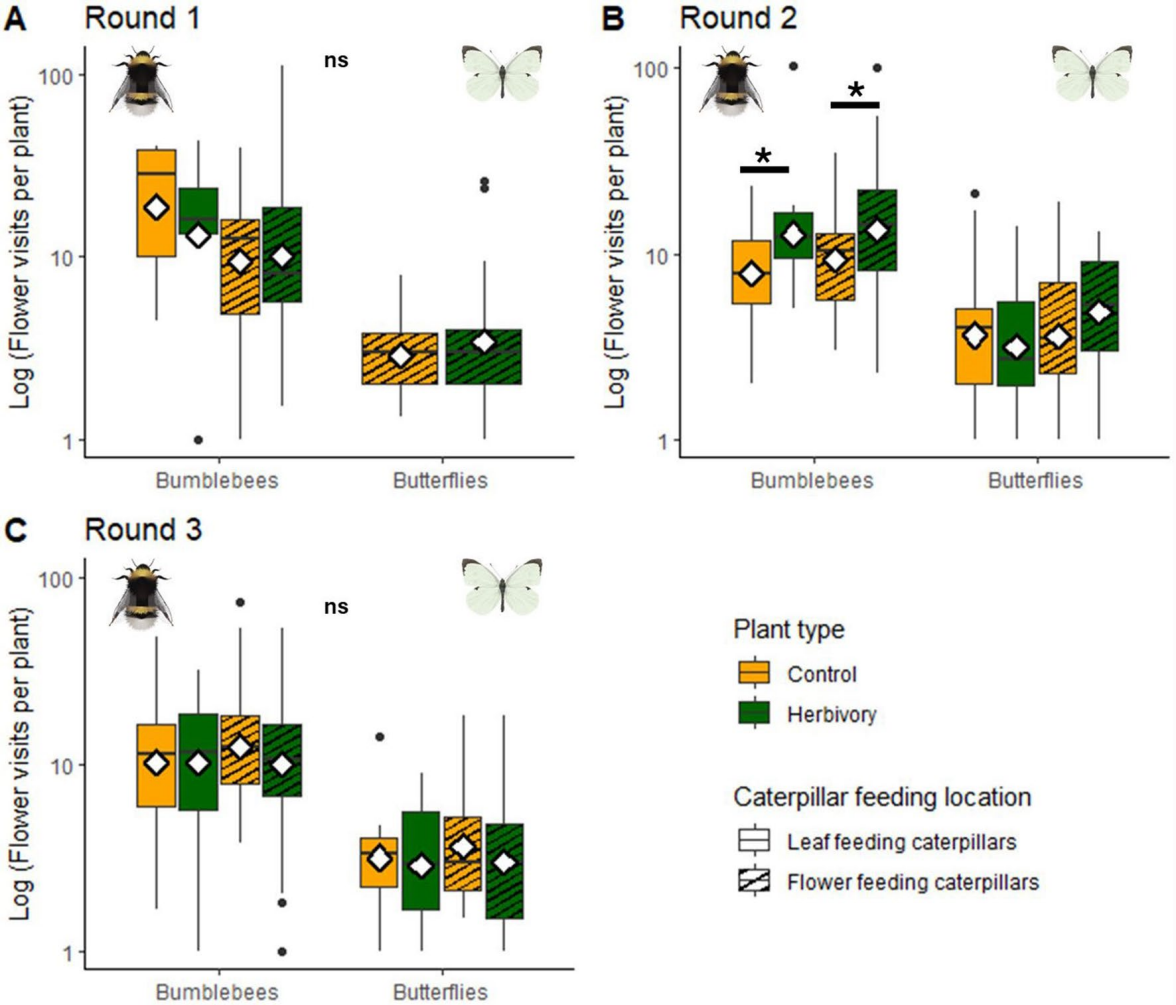
Number of flowers visited per plant with or without herbivory, by bumblebees and butterflies*. A–C* Experimental rounds 1–3. Yellow boxes indicate control treatments for folivory (blank) and florivory (striped). Green boxes indicate herbivory treatments of folivory (blank) and florivory (striped). Significance code: *p < 0.05

Bumblebees visited more flowers per plant than butterflies: on average 14.79 (± 11.94), compared to 5.05 (± 4.29) on average for a butterfly plant visit (LMM, Pollinator main effect, *χ*^2^ = 244.32, *df* = 1, *p* < 0.001, Figure S2c). The effects of herbivory on plant visits differed per experimental round (Treatment * Experimental round interaction, LMM, *χ*^2^ = 9.65, *df* = 2, *p* = 0.008). Caterpillar presence had the largest effect on the number of flower visits by bumblebees in the second experimental round. In this second round, bumblebees visited more flowers on plants with caterpillars, compared to control plants (Supplementary Table S6, Fig. 4). Visit numbers increased on both plants with folivorous caterpillars (Tukey, *p* = 0.04), and florivorous caterpillars (Tukey, *p* = 0.02). In the first and third experimental rounds, the number of bumblebee visits to plants with caterpillars remained unchanged compared to control, under both folivory and florivory conditions. The number of flower visits by butterflies remained equal in presence or absence of caterpillars regardless of their feeding location or experimental round.

#### Time spent per flower

**Fig. 5:**
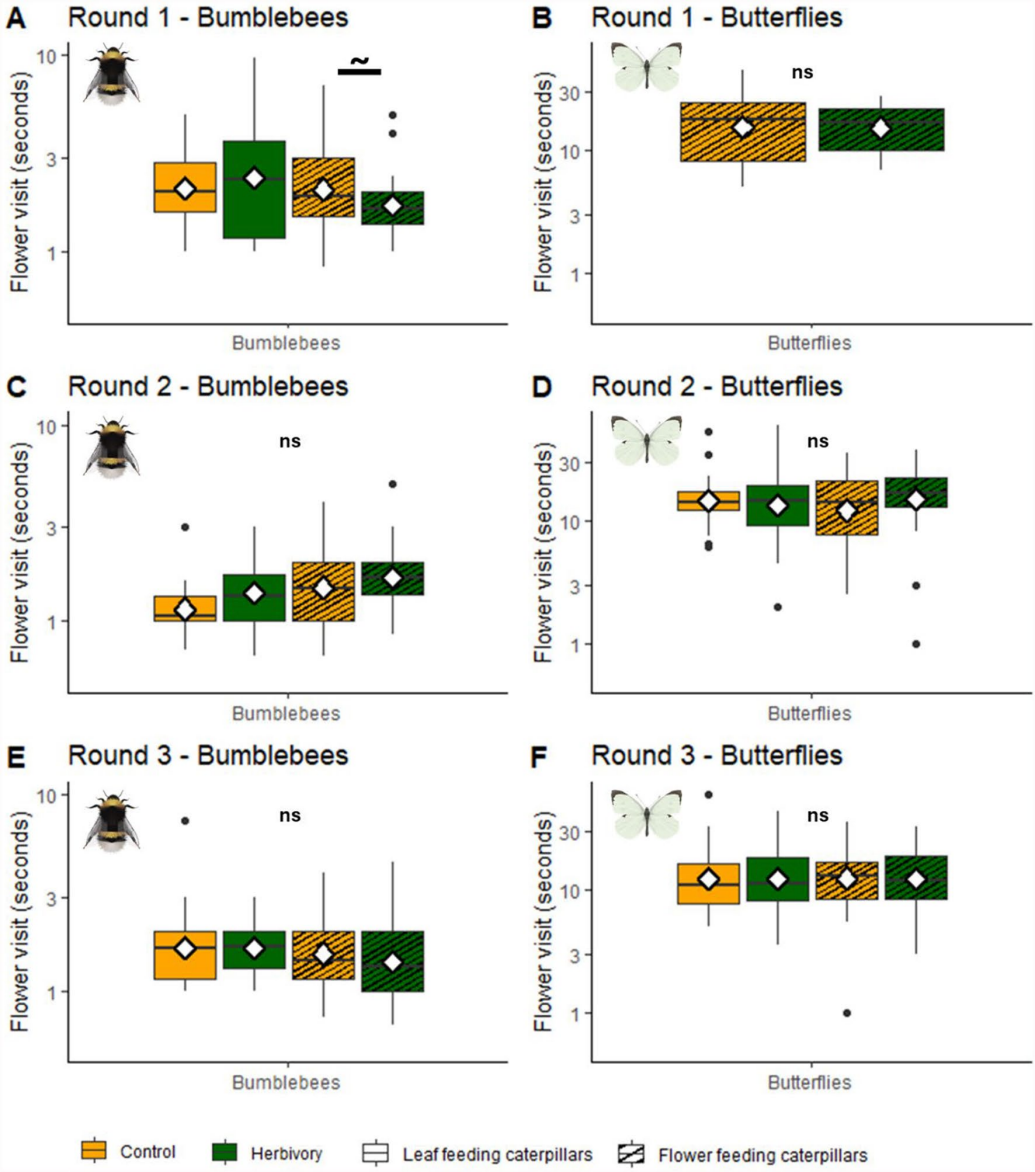
Time spent per flower visit per plant with or without herbivory, by bumblebees and butterflies*. A***, B** First experimental round. **C, D** Second experimental round. **E, F** Third experimental round Yellow boxes indicate control treatments for folivory (blank) and florivory (striped). Green boxes indicate herbivory treatments of folivory (blank) and florivory (striped).Significance code: ~ *p* < 0.1

**Fig. 6:**
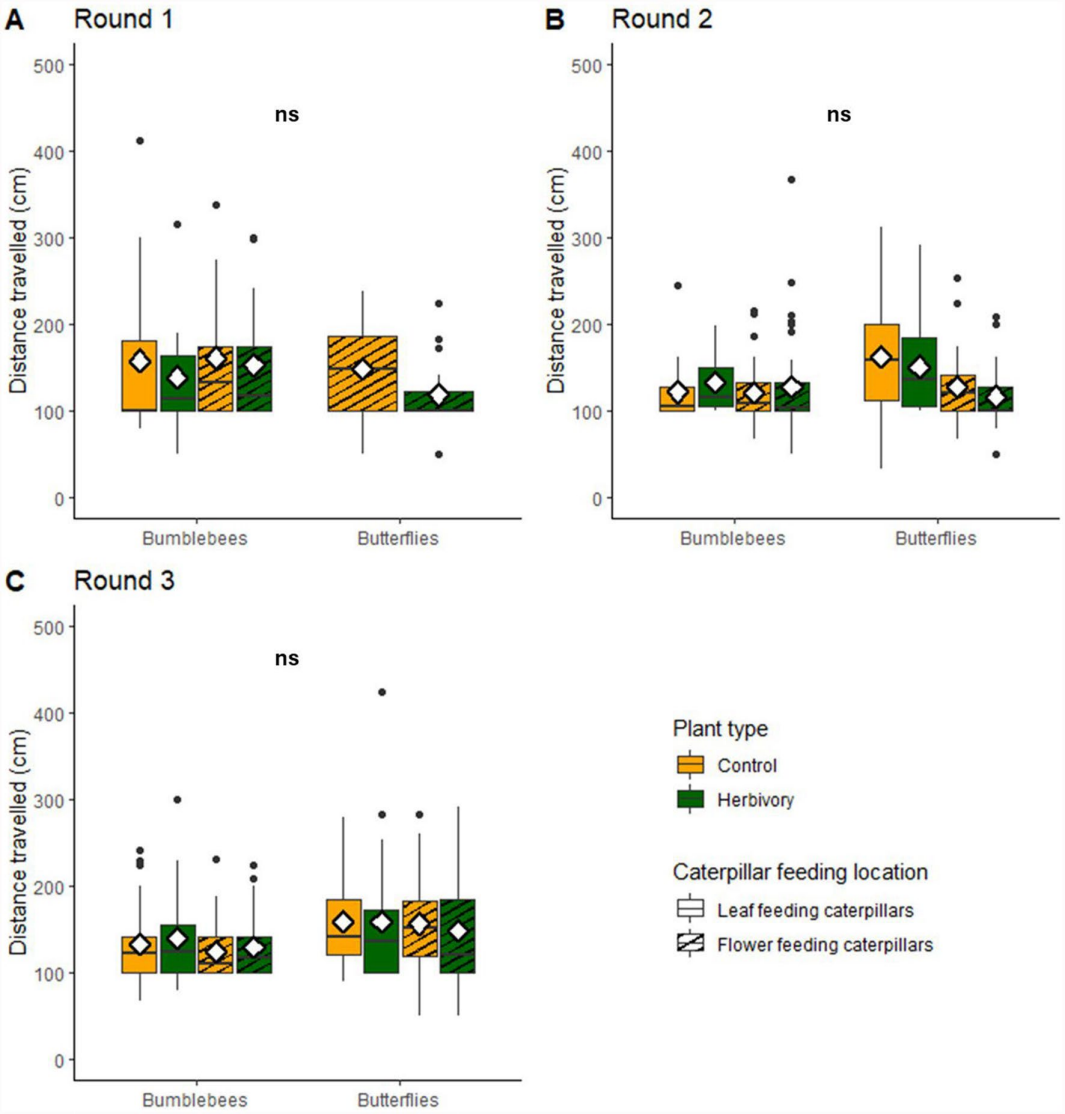
Distance travelled to the next plant after visiting a plant with or without herbivory, by bumblebees and butterflies*. A–C* Experimental rounds 1–3. Yellow boxes indicate control treatments for folivory (blank) and florivory (striped). Green boxes indicate herbivory treatments of folivory (blank) and florivory (striped)

Bumblebees on average spent less time per flower visit than butterflies (Welch’s *t* test, *t* = − 18.272, *df* = 148.08, *p* < 0.001, Figure S2d); bumblebee visits lasted 2.02 s (± 1.96 s), whereas butterfly visits lasted 17.04 s (± 9.57 s). Effects of experimental round, caterpillar feeding location and herbivory treatment on time spent per flower were analysed separately for bumblebees and butterflies (see Methods). For bumblebees, the time spent on flowers of plants with or without herbivores depended on the experimental round (LMM, *χ*^2^ = 20.66, *df* = 2, *p* < 0.001, Fig. 5), as well as the interactions between experimental round and treatment (LMM, *χ*^2^ = 6.54, *df* = 2, *p* = 0.04), and between experimental round and caterpillar feeding location (LMM, *χ*^2^ = 7.87, *df* = 2, *p* = 0.02). Post hoc testing revealed only a marginally significant decrease in flower visit duration in response to florivory compared to control (Tukey, *p* = 0.08), in the first experimental round. Butterfly flower visit time was not affected by herbivory (LMM, treatment main effect, *χ*^2^ = 0.12, *df* = 1, *p* = 0.73, Fig. 6), caterpillar feeding location (LMM, main effect, *χ*^2^ = 0.4, *df* = 1, *p* = 0.84) nor experimental round (LMM, main effect, *χ*^2^ = 2.45, *df* = 2, *p* = 0.30).

### Spatial behaviour

**Fig. 7:**
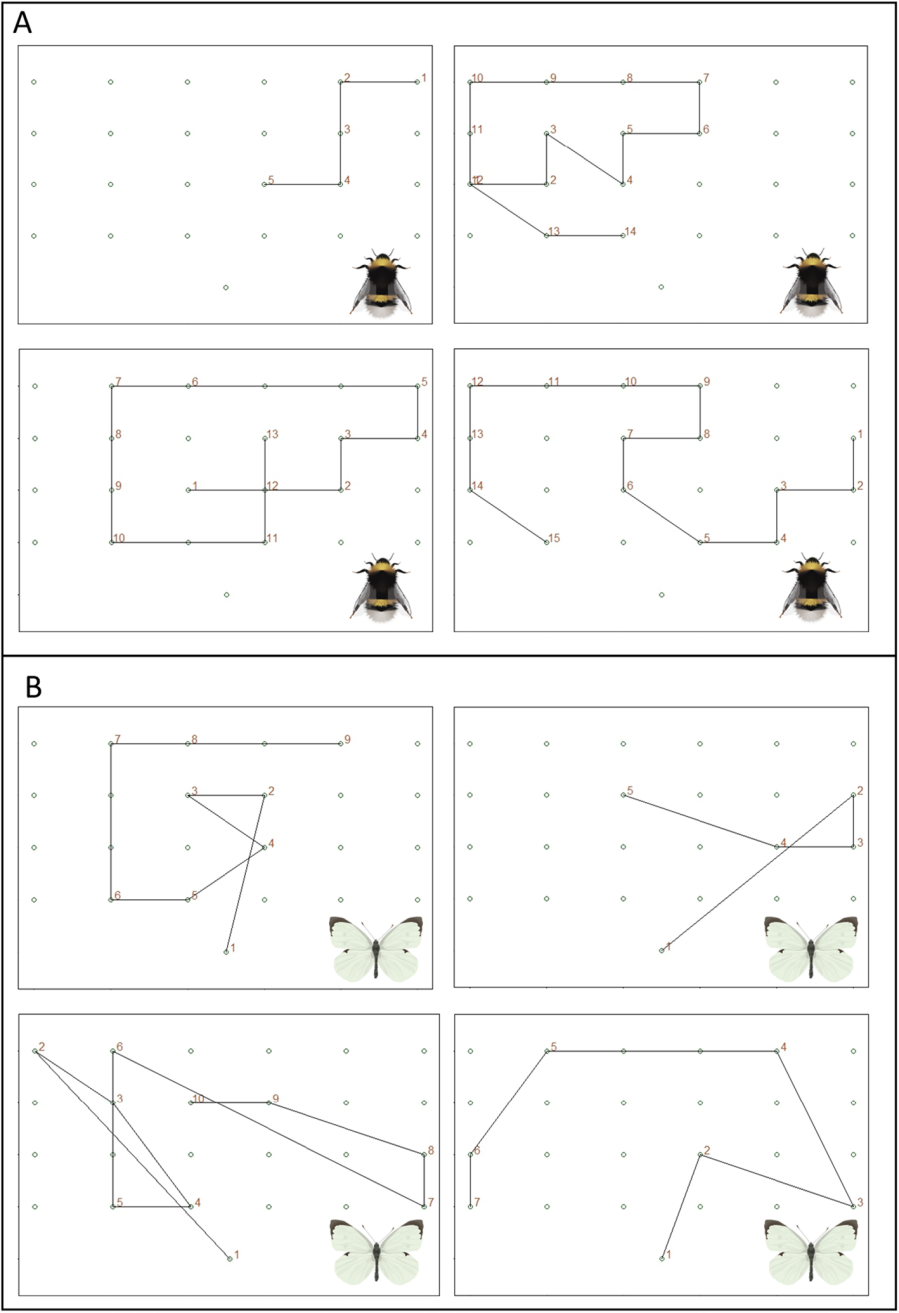
Flight paths by bumblebees (**A**) and butterflies (**B**). Visualization of the trajectory of plant visits during the observation, of four individuals per species. Circles indicate individual plants. Numbers indicate plant visits in order. In **B** number 1 shows the butterfly release point

Butterflies traveled longer distances between plants than bumblebees (Welch’s *t* test, *t* = − 10.072, *df* = 282.59, *p* < 0.001, Figure S2E). The distance between plants in the experiment was 1 m, or 1.41 m diagonally. Bumblebees traveled on average 1.36 (± 0.52) meters, indicating that they primarily visited neighboring plants. Butterflies frequently traveled longer distances, covering on average 2.11 (± 0.83) meters between plant visits. Despite the longer distances travelled by butterflies, the overall distance each pollinator covered during a 10-min observation was similar, due to the higher frequencies of plant visits by bumblebees. Bumblebees travelled on average 12.00 (± 8.05) meters, compared to 12.19 (± 8.50) meters for butterflies (Welch’s *t* test, *t* = − 1.159, *df* = 159.03, *p* = 0.874, Figure S2F). The distance traveled to the next visit was unaffected by whether the previous visit was to a plant with or without caterpillars, regardless of the caterpillar's feeding location (GLMM, treatment main effect, *χ*^2^ = 0.06, *df* = 1, *p* = 0.80, Fig. 6). Some examples of travel paths of butterflies and bumblebees are depicted in Fig. 7.

## Discussion

This study investigated behavioural responses of pollinators to herbivory, and how such effects are mediated by pollinator identity and type of herbivore damage. We found that bumblebees and butterflies have unique behavioural responses to herbivory on plants. Bumblebees visited damaged and undamaged plants at equal rates, but spent more time and visited more flowers on plants with herbivory. This response was present in only one of three experimental rounds, suggesting an interaction between herbivory and another, unknown, environmental factor in shaping bumblebee visitation behaviour. In contrast, butterfly behaviour was affected at the visitation stage, but not after choices were made. Specifically, butterflies sometimes preferred to visit plants with florivory, and sometimes avoided them, depending on experimental round. We also found that visitation behaviours differed between bumblebees and butterflies overall, consistent with other studies of pollinator behaviour across diverse pollinator guilds (Brunet and Sweet 2006; Lysenkov 2009).

The explanation for the different behavioural responses by bumblebees and butterflies may lie in their different foraging strategies. Bumblebees are mainly sensitive to rewards, and evaluate reward quality by tasting, rather than through olfactory cues (Ruedenauer et al. 2015, 2016). This could explain why bumblebees altered their behaviour during plant visits, but not in the choice of plant to visit. In contrast, *Pieris* butterflies are responsive to volatiles that result from glucosinolate breakdown upon herbivore feeding (van Loon et al. 1992). Alternatively, for butterflies the presence of their conspecifics on the plant may hold a different meaning, related to reproduction rather than foraging behaviour. These volatiles serve as cues for female *P. brassicae* adults to initiate egg-laying (van Loon et al. 1992). While our study only used males, the response may either be conserved or act as a cue in mate finding. Earlier work identified that honeybees also do not show a preference for plants with or without caterpillars either, while other wild pollinators tended to visit plants without caterpillars more often (Scopece et al. 2019). Thus, our study confirms that pollinator identity matters in behavioural responses to insect herbivory.

Other studies have shown that plant disease or defence against herbivores can alter pollen dispersal through changes in pollinator behaviour (Koupilová et al. 2021; Scopece et al. 2019). For example, pollinators avoid plants infected with anther smut fungus, altering pollen flow between other plant individuals in the same patch (Koupilová et al. 2021). In our study, travel distance between plants was not affected by herbivory, regardless of caterpillar feeding location. As bumblebees did not alter their plant choices, the distance covered between plant visits was unlikely to be affected by our herbivory treatment. Generally, bumblebees visited neighboring plants, optimizing their foraging strategy for maximum pollen and nectar collection by reducing travel time between sources (Pyke 1980). Butterflies generally travelled larger distances between plants, and in a seemingly more random pattern. As our control and induced plants were placed in a chessboard pattern with equal distance to the next plant with or without caterpillars, average distance travelled may have remained unchanged by encounter with a previous plant treatment. In more natural situations with a more patchy lay-out of plants with and without herbivores, the effects of plant preference may be more clearly reflected in travel distances. Additionally, our experimental set-up was limited by the 12 × 12 m tent size. Differences in distance travelled may be more pronounced on larger spatial scales.

We found that butterfly and bumblebee sensitivity to herbivory changed as herbivores switched from damaging leaves to damaging flowers. Neither butterflies nor bumblebees significantly changed their behaviour in response to folivorous herbivory. This could mean that at this stage, they did not perceive a difference between control or herbivory plants. Herbivory by *P. brassicae* on *B. rapa* can decrease floral volatile emission, rendering plants less attractive to *B. terrestris* (Schiestl et al. 2014). We did not observe a decrease in bumblebee attraction upon folivory, which may indicate that our induction was not strong enough to elicit a plant response relevant to bumblebees. Our induction only involved eight caterpillars, which is less than in other studies (Bruinsma et al. 2014; Chrétien et al. 2018) or the natural situation, where *P. brassicae* butterflies lay clutches of 10–100 eggs and caterpillars feed gregariously (Davies and Gilbert 1985). The responses in pollinator behaviour became more apparent as the caterpillars started feeding on flowers. While the eight folivores did not consume large amounts of leaf tissue, due to their small size, in their larger folivores stages the eight caterpillars significantly damaged the floral branches. This could explain the increased sensitivity in pollinator responses. Additionally, the observed changes in pollinator response to florivory may have resulted from a stronger (Farré-Armengol et al. 2015) or different cue emitted by the flowers (Chrétien et al. 2018, 2021), or from a difference in reward quality or quantity (Rusman et al. 2019a, b; Schiestl et al. 2014; Stevenson et al. 2017).

Both general foraging differences between bumblebees and butterflies and their response to herbivory can have consequences for plant reproduction. Our study shows that bumblebees generally visit many plants and flowers in a short amount of time, indicating that they contribute greatly to pollen transfer. However, their many visits to flowers on the same plant may in the case of self-incompatible *Brassica rapa* result in pollen loss or clogging of stigmas; an effect that is increased by a higher number of flower visits on plants with herbivory. Butterflies visit fewer plants in general and may therefore contribute less to the overall mating opportunities for *Brassica rapa.* Butterflies did alter their plant preference in response to herbivory, which may have severe consequences for plant reproduction of the plants that are no longer visited. The direction of this effect was variable in our study, which suggests that the plants response to herbivory interacts with an environmental factor that might dampen or increase the effects of herbivory on pollinator behaviour. Weather conditions such as temperature and drought are both known to affect pollinator behaviour (Höfer et al. 2021).

In conclusion, this study contributes to our understanding of the dynamics of plant reproduction in plant-antagonist-mutualist interactions. It demonstrates that behavioural differences between butterflies and bumblebees have the potential to influence spatial movement patterns of pollen. Attracting a more diverse pollinator community may thus enhance diversity in received pollen, enabling plants to evolve mechanisms to select their preferred mate from the genetically diverse deposited pollen (Huth and Pellmyr 2000). This, in turn, results in increased gene flow at population level, allowing plants to adapt to environmental changes (Barrett and Harder 2017). *Brassica rapa* has a very diverse pollinator community, which also includes honeybees, solitary bees, hoverflies and other species of bumblebees and butterflies (Rader et al. 2009). This diverse community might help *B. rapa* to mitigate the effects of insect attacks. For example, a decrease in butterfly visits to plants with herbivory may have negative consequences for pollen pick-up and receipt, but this effect could be mitigated by the unaltered visitation behaviour of bumblebees. It would be interesting to see future studies include measurements of pollination success, such as seed production. Connecting the observed changes in pollinator behaviour to plant fitness can provide more insights into the evolutionary consequences of plasticity in interactions with pollinators. For future research, incorporating additional members of the pollinator community, and quantifying the effects of altered pollinator behaviour on plant reproductive success will help to further unravel the consequences of herbivory for pollen transfer and gene flow. 

## Supplementary Information

Below is the link to the electronic supplementary material.

Supplementary file1 (DOCX 1338 kb)

## Data Availability

The datasets used and/or analysed during the current study are available from the corresponding author on reasonable request.
