# Supplementary material for: Insect herbivory differentially affects the behaviour of two pollinators of Brassica rapa
Source: Oecologia. 2025 Aug 14;207(9):143. doi: 10.1007/s00442-025-05777-2 (PMC12354498; doi:10.1007/s00442-025-05777-2)
Supplement: Supplementary file 1 — Supplementary file1 (DOCX 1338 kb) [file 442_2025_5777_MOESM1_ESM.docx]

# Supplementary Figures


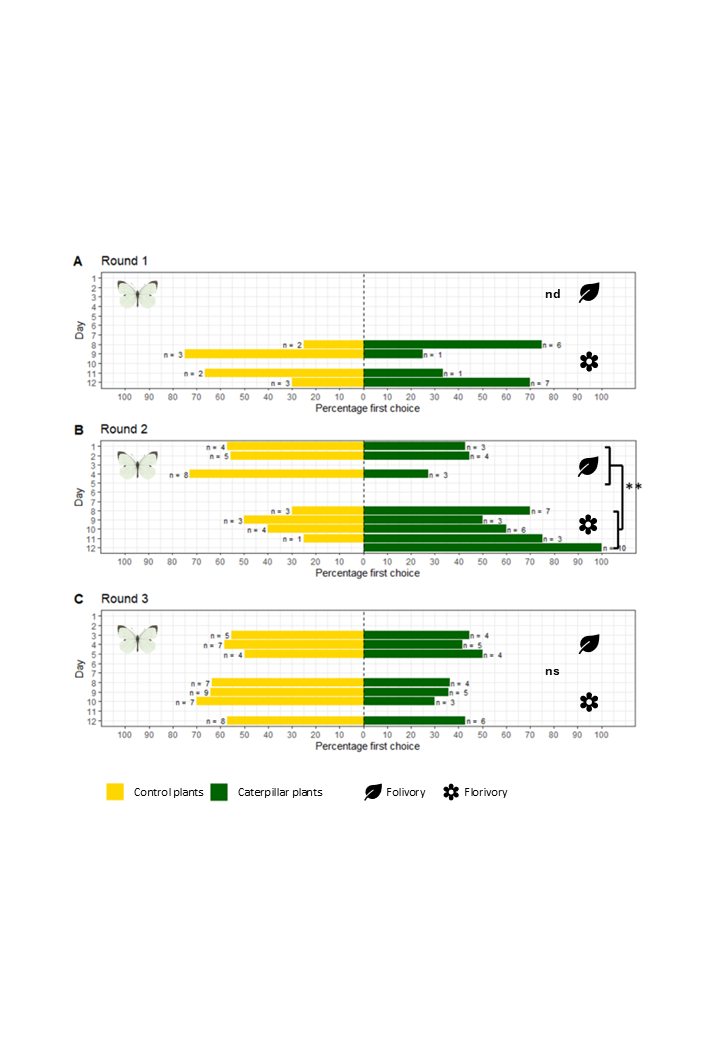


**Figure S1: First choice over time of *Pieris brassicae* butterflies during three experimental rounds.** First preference of each individual plotted for each day of the three two-week experimental rounds (A-C). Day 1 marks the day of induction of plants with *Pieris brassicae* caterpillars. N = number of individuals. Yellow boxes indicate control treatment. Green boxes indicate herbivory treatment. Significance codes ** p < 0.005, nd = no data, ns = not significant.


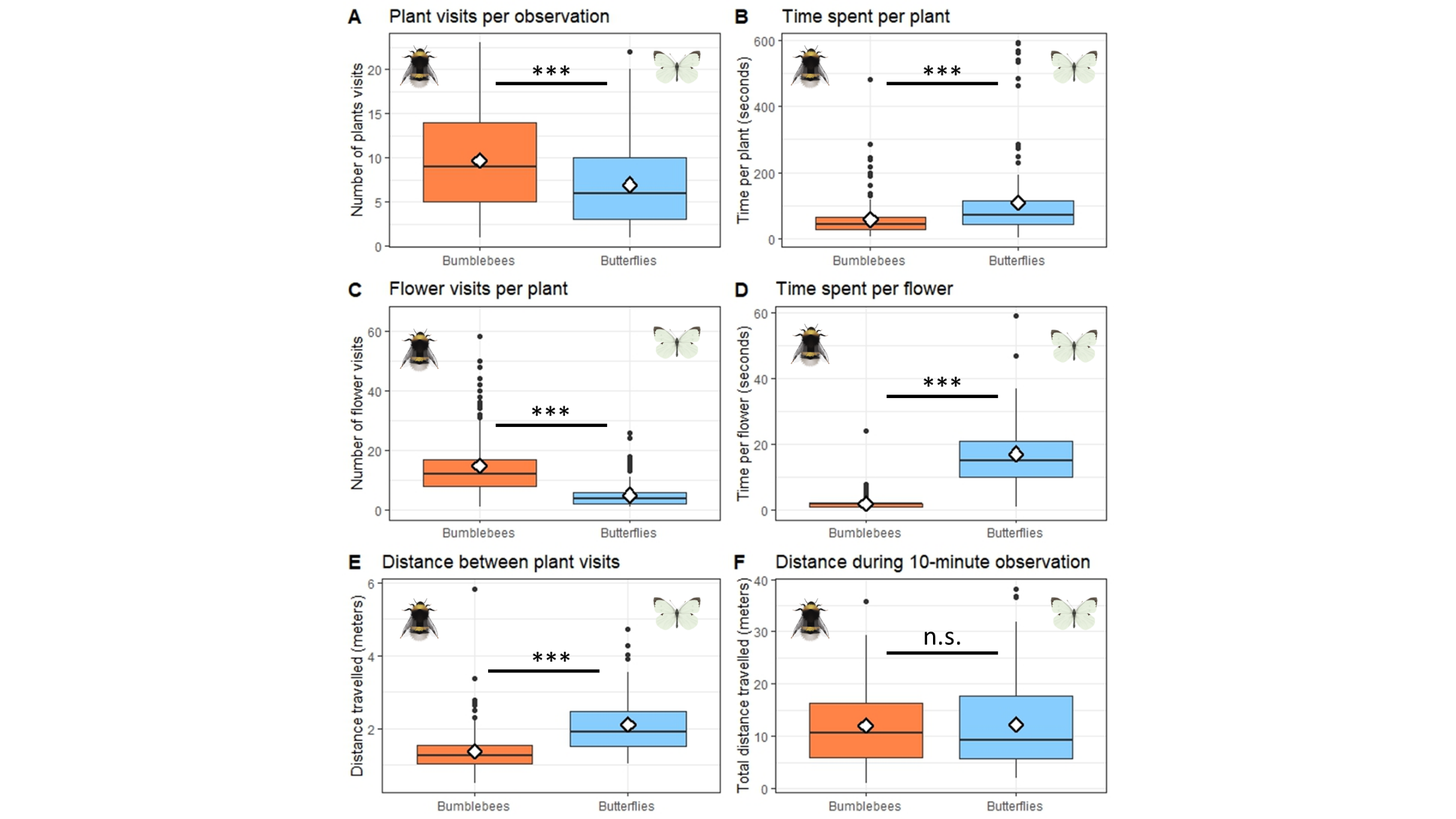


**Figure S2: Behavioural differences between *Bombus terrestris* bumblebees and *Pieris brassicae* butterflies*.* A** Number of plants visited per 10-minute observation. **B.** Average time in seconds spent per plant per observation. **C.** Average number of flowers visited per plant per observation. **D.** Average time spent per flower visit per observation. **E.** Average distance travelled per observation between subsequent plants. **F.** Distance travelled per 10-minute observation. Significance codes: *** p < 0.001, n.s. = not significant,

**
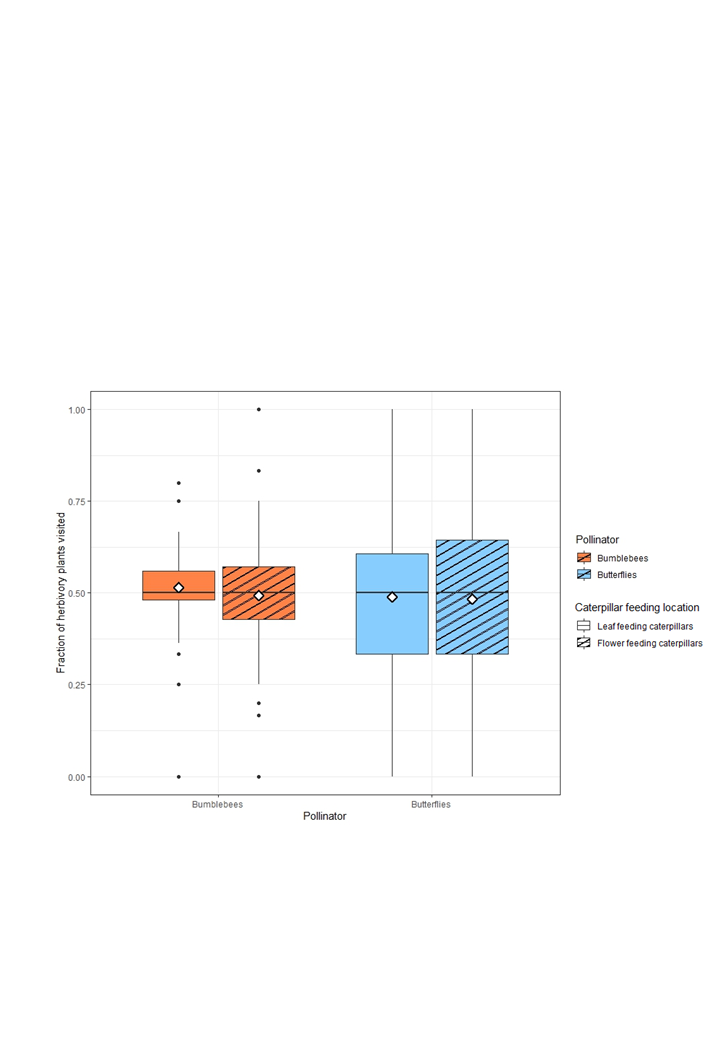
**

**Figure S3: Fraction of herbivore-treated plants visited per observation by the bumblebee (*Bombus terrestris,* orange boxes) and butterfly (*Pieris brassicae,* blue boxes).** Fractions are presented per herbivory treatment. Blank boxes indicate folivory, striped boxes indicate florivory. Diamonds indicate the mean scores.

## Supplementary Tables

**S1. Binomial test results for first choice of *Pieris brassicae* in each week of the experimental rounds with values in bold indicating significant preference.**

| **Week** | **Round** | **First choice for herbivory** | **Number of observations** | **Percentage** | **p-value** |
| --- | --- | --- | --- | --- | --- |
| 1 | 2 | 10 | 27 | 37% | 0.248 |
| 1 | 3 | 13 | 29 | 45% | 0.711 |
|  |  |  |  |  |  |
| 2 | 1 | 15 | 25 | 60% | 0.424 |
| 2 | 2 | 29 | 40 | 73% | **0.006** |
| 2 | 3 | 18 | 49 | 37% | 0.085 |

**S2. Number of plants visited per observation for each pollinator**

| **Pollinator** | **Plants visited per observation** (mean ± sd) | **Control plants visited per observation** (mean ± sd) | **Herbivory plants visited per observation** (mean ± sd) |
| --- | --- | --- | --- |
| Bumblebee (*Bombus terrestris*) | 9.69 (± 5.87) | 4.96 (± 3.23) | 4.73 (± 2.92) |
| Butterfly  (*Pieris brassicae*) | 6.93 (± 4.93) | 3.64 (± 2.94) | 3.30 (± 2.60 |

**S3. Time spent (in seconds) on plant visitation per pollinator and treatment per observation**

| **Pollinator** | **Total time on plants** (mean ± sd) | **Total time on control** (mean ± sd) | **Total time on herbivory**  (mean ± sd) | **Average time on control** (mean ± sd) | **Average time on herbivory** (mean ± sd) |
| --- | --- | --- | --- | --- | --- |
| Bumblebee  (B*ombus terrestris*) | 515.23 (± 134.19) | 250.88 (± 132.32) | 264.35 (± 132.20) | 77.91 (± 106.38) | 74.78 (± 75.66) |
| Butterfly  (*Pieris brassicae*) | 526.23 (± 108.73) | 251.42 (± 184.49) | 274.81 (±194.14) | 102.36 (± 138.75) | 123.32 (± 154.18) |

**S4. Fraction of plants with herbivory visited per individual observation**

| **Pollinator** | **Herbivory type** | **Fraction herbivory plants** (mean ± sd) |
| --- | --- | --- |
| Bumblebee (*Bombus terrestris*) | Total | 0.50 (± 0.15) |
|  | Folivory | 0.51 (± 0.12) |
|  | Florivory | 0.49 (± 0.16) |
| Butterfly (*Pieris brassicae*) | Total | 0.48 (± 0.26) |
|  | Folivory | 0.49 (± 0.24) |
|  | Florivory | 0.48 (± 0.27) |

**S5. Time spent per plant visit by bumblebees and butterflies on plants with and without herbivory**

| **Round** | **Herbivory type** | **Pollinator** | **Average time spent per herbivory plant** (seconds, mean ± sd) | **Average time spent per control plant** (seconds, mean ± sd) |
| --- | --- | --- | --- | --- |
| All | All | Bumblebee | 61.39 (± 73.23) | 50.77 (± 45.50) |
| 1 | Folivory | Bumblebee | 77.33 (± 73.53) | 90.58 (± 64.39) |
| 1 | Florivory | Bumblebee | 76.52 (± 108.27) | 52.88 (± 39.71) |
| 2 | Folivory | Bumblebee | 58.00 (± 100.67) | 28.33 (± 17.27) |
| 2 | Florivory | Bumblebee | 65.86 (± 59.43) | 40.88 (± 26.62) |
| 3 | Folivory | Bumblebee | 45.08 (± 33.33) | 45.96 (± 38.19) |
| 3 | Florivory | Bumblebee | 54.04 (± 61.29) | 59.81 (± 0.44) |
| All | All | Butterfly | 98.90 (± 120.02) | 84.92 (± 90.00) |
| 1 | Folivory | Butterfly | - | - |
| 1 | Florivory | Butterfly | 119.84 (± 159.42) | 77.79 (± 61.74) |
| 2 | Folivory | Butterfly | 112.70 (± 154.81) | 103.14 (± 101.18) |
| 2 | Florivory | Butterfly | 111.04 (± 70.66) | 81.24 (± 62.42) |
| 3 | Folivory | Butterfly | 62.20 (± 69.22) | 73.57 (± 104.82) |
| 3 | Florivory | Butterfly | 89.47 (± 124.85) | 86.27 (± 97.39) |

**S6. Average number of flowers visited per plant visit by bumblebees and butterflies on plants with and without herbivory**

| **Round** | **Herbivory type** | **Pollinator** | **Average number of flowers visited per herbivory plant** (mean ± sd) | **Average number of flowers visited per control plant**  (mean ± sd) |
| --- | --- | --- | --- | --- |
| All | All | Bumblebee | 15.77 (± 16.74) | 13.83 (± 11.56) |
| 1 | Folivory | Bumblebee | 17.92 (± 14.02) | 23.67 (± 15.11) |
| 1 | Florivory | Bumblebee | 17.93 (± 25.65) | 13.47 (± 10.82) |
| 2 | Folivory | Bumblebee | 16.40 (± 21.09) | 9.50 (± 6.13) |
| 2 | Florivory | Bumblebee | 18.14 (± 17.44) | 11.55 (± 7.58) |
| 3 | Folivory | Bumblebee | 12.54 (± 8.08) | 14.21 (± 12.11) |
| 3 | Florivory | Bumblebee | 13.11 (± 10.45) | 15.19 (± 14.13) |
| All | All | Butterfly | 4.81 (± 4.41) | 4.39 (± 3.88) |
| 1 | Folivory | Butterfly | - | - |
| 1 | Florivory | Butterfly | 5.53 (± 7.15) | 3.29 (± 2.05) |
| 2 | Folivory | Butterfly | 4.35 (± 3.66) | 5.05 (± 5.14) |
| 2 | Florivory | Butterfly | 6.11 (± 3.62) | 5.10 (± 4.45) |
| 3 | Folivory | Butterfly | 3.70 (±2.57) | 3.57 (± 2.50) |
| 3 | Florivory | Butterfly | 4.32 (± 4.28) | 4.54 (± 3.94) |

**S7. Average time spent per flower per plant visit by bumblebees and butterflies on plants with and without herbivory**

| **Round** | **Herbivory type** | **Pollinator** | **Average time spent per flower on herbivory plant** (seconds, mean ± sd) | **Average time spent per flower on control plant**  (seconds, mean ± sd) |
| --- | --- | --- | --- | --- |
| All | All | Bumblebee | 1.87 (± 1.52) | 1.94 (± 2.34) |
| 1 | Folivory | Bumblebee | 2.92 (± 2.54) | 2.08 (± 1.24) |
| 1 | Florivory | Bumblebee | 1.85 (± 0.95) | 2.47 (± 1.32) |
| 2 | Folivory | Bumblebee | 1.40 (± 0.75) | 1.17 (± 0.51) |
| 2 | Florivory | Bumblebee | 1.82 (± 0.81) | 1.60 (± 0.83) |
| 3 | Folivory | Bumblebee | 1.75 (± 0.74) | 1.79 (± 1.25) |
| 3 | Florivory | Bumblebee | 1.91 (± 2.27) | 2.23 (± 4.27) |
| All | All | Butterfly | 16.11 (± 9.59) | 15.56 (± 9.91) |
| 1 | Folivory | Butterfly | - | - |
| 1 | Florivory | Butterfly | 16.74 (± 6.77) | 19.21 (± 12.12) |
| 2 | Folivory | Butterfly | 17.09 (± 13.43) | 16.71 (± 10.53) |
| 2 | Florivory | Butterfly | 17.75 (± 8.73) | 14.67 (± 8.62) |
| 3 | Folivory | Butterfly | 14.80 (± 10.08) | 15.26 (± 12.40) |
| 3 | Florivory | Butterfly | 14.53 (± 8.37) | 14.34 (± 7.76) |
